# Evaluating the Effectiveness of Coxal Bone Measurements for Sex Estimation via Machine Learning

**DOI:** 10.3390/biology14070866

**Published:** 2025-07-17

**Authors:** Diana Toneva, Silviya Nikolova, Gennady Agre, Nevena Fileva, Georgi Milenov, Dora Zlatareva

**Affiliations:** 1 Institute of Experimental Morphology, Pathology and Anthropology with Museum, Bulgarian Academy of Sciences, 1113 Sofia, Bulgaria; sil_nikolova@abv.bg; 2 Institute of Information and Communication Technologies, Bulgarian Academy of Sciences, 1113 Sofia, Bulgaria; gennady.agre@iict.bas.bg; 3 Faculty of Medicine, Medical University of Sofia, 1431 Sofia, Bulgaria; nfileva@medfac.mu-sofia.bg (N.F.); g.milenov@medfac.mu-sofia.bg (G.M.); dorazlat@medfac.mu-sofia.bg (D.Z.)

**Keywords:** coxal bone, sex, measurements, machine learning, computed tomography

## Abstract

The pelvis is the most dimorphic part of the human skeleton, primarily because of its involvement in the birth process. Many sexually dimorphic traits are concentrated in the coxal bones, which form the larger part of the birth canal. The present study aimed to assess the sex differences in coxal bone size and to develop machine learning (ML) models for sex estimation based on coxal bone measurements. The sample included abdominal computed tomography scans of 276 adult Bulgarians. Three-dimensional models of the pelves were generated using InVesalius. The three-dimensional coordinates of 34 landmarks located on the right and left coxal bones were collected in MeshLab. Based on the landmark coordinates, various measurements characterizing the coxal bones were calculated. The coxal bone dimensions were tested for significant differences with respect to sex, age, and laterality. Support Vector Machines and logistic regression were employed to train models for sex estimation. The results demonstrate strong sexual dimorphism in coxal bone dimensions along with some bilateral and age-related differences. The trained ML models classify male and female bones with very high accuracy, ranging between 95% and 100%.

## Simple Summary

Sex estimation plays a pivotal role in the reconstruction of the biological profile from skeletal remains across various branches of anthropological science. The human pelvis is a key structure in this process, as its morphology differs substantially between males and females due to the distinct demands of pregnancy and childbirth in females. Many studies have examined sex differences in the size and shape of the coxal bones, which form the major part of the pelvis; however, only a few have applied machine learning algorithms for this purpose. The present study applied such methods to evaluate the potential of coxal bone measurements and trained models to correctly classify male and female pelves.

## 1. Introduction

Sex estimation based on human skeletal remains is essential in a range of anthropological fields, supporting both identification and population-level analyses. Estimating sex from skeletal remains provides valuable biological information that supports forensic scientists in reconstructing the biological profile. Accurate sex estimation can significantly aid investigations in criminal cases, disaster victim identification, and mass grave examination by contributing to personal identification and directing the efficient allocation of resources and search efforts. Accurate sex estimation not only facilitates the identification of unknown remains in forensic cases but also contributes to biological anthropology, where analyzing sex differences and the accuracy of sex estimation models provides insights into the sexual dimorphism expressed within the studied population. In bioarcheology, sex estimation is important for reconstructing the demographic structure of past populations and for associating burial artifacts and practices with biological sex.

The pelvis is considered the most dimorphic part of the human skeleton, likely due to the different morphological demands in males and females related to pregnancy and childbirth. In males, the pelvis is narrower and more funnel-shaped, whereas in females it is wider and more circular. However, pelvic morphology is also required to support locomotion in both sexes. Particularly in females, it must balance the antagonistic selective pressures favoring maternal bipedal locomotion and neonatal encephalization, a compromise often referred to as the ‘obstetric dilemma’ [1,2,3]. Thus, the sexual dimorphism in pelvic morphology is determined not only by body size differences but also by the requirements of offspring development and delivery, which are facilitated by the pelvic bones and ligaments. Since the human pelvis is composed of several bones (i.e., the left and right coxal bones, sacrum, and coccyx), it is expected that each of these bones will exhibit strong dimorphic traits. The coxal bones form the largest part of the birth canal, and that is why they have been the most frequently examined.

The sustained focus on the coxal bones for sex estimation has led to numerous studies examining the sex differences manifested in their size and shape. Most of these studies have been conducted on dry bones [4,5,6,7,8,9,10,11,12,13,14,15,16,17,18,19,20,21,22,23,24,25,26,27,28], with some of them incorporating the use of digitizers [29,30,31]. In recent years, however, there has been a growing prevalence of three-dimensional (3D) imaging techniques, such as computed tomography (CT) [21,26,32,33,34,35,36] and surface scanning [36]. A recent study demonstrated that dry bones, 3D surface scans, and CT images can be used interchangeably for applying both non-metric and metric pelvic sex estimation techniques [37]. The ability to collect both metric and non-metric data from contemporary individuals of known sex and age makes CT imaging a preferred modality for developing sex estimation methods.

Numerous morphoscopic studies have been conducted to evaluate the sex-discriminating power of various visually assessed traits of the coxal bone, either individually or in combination, such as the shape of the greater sciatic notch, the presence of the ischiopubic ridge, the ventral arc, the subpubic concavity of the pubic bone, the orientation of the ischial tuberosity, the shape of the obturator foramen, and the presence of the preauricular sulcus [4,8,10,13,14,17,19,24,26]. However, many studies have focused on metric features of the coxal bones, with the number of measurements ranging from just a few to larger sets containing more than forty variables [5,6,7,8,9,12,16,21,22,25,28,32,33,36,38]. To estimate sex from coxal bone measurements, various classification methods have been applied in morphometric studies. These methods span traditional statistical analyses such as linear discriminant analysis (DA) and binary logistic regression (LR) [6,7,9,12,16,21,25,28,32,39], as well as more recent machine learning (ML) and deep learning (DL) approaches [23,33,34,35,38]. Additionally, some studies have utilized landmark-based geometric morphometrics [11,15,17,18,26,29,30,31] and outline-based methods, such as Fourier analysis [20,24,27], to investigate sex differences in the shape and size of the coxal bone or its specific regions.

ML is a technique that enables computers to learn from data, identify patterns, and make decisions or predictions without being explicitly programmed to perform these tasks. It is widely applicable in fields such as medicine and image analysis, where structured and unstructured datasets can be used to train models for tasks such as classification, prediction, and detection. The relatively few ML studies focused on sex classification based on the coxal bone have used different features as input variables for training the models. Nikita and Nikitas [23] applied ML algorithms to score data derived from visual traits, while Coelho and Curate [38] and Secgin et al. [33] trained models on metric coxal bone data. In contrast, Cao et al. [34] utilized convolutional neural networks to predict sex from two-dimensional images of various coxal bone regions, and Epain et al. [35] employed autoencoders to reconstruct and classify 3D meshes of paired coxal bones.

The present study aims to assess the sex differences in coxal bone measurements and to identify the most dimorphic metric features by applying ML techniques and developing models for sex estimation.

## 2. Materials and Methods

The sample included abdominal CT scans of 276 adult Bulgarians (136 males and 140 females). The mean age of the males was 56.5 ± 14.8 years (range: 19–83 years) and that of the females was 56.7 ± 13.8 years (range: 19–88 years). The images were generated using a medical CT system (Toshiba Aquilion 64). The scanning protocol was as follows: 32 × 0.5 mm detector configuration, tube current ranging from 100 to 392 mA, tube voltage of 120 kV, and exposure time of 500 ms. The images were reconstructed with a 512 × 512 reconstruction matrix, a slice thickness of 2 mm, a reconstruction interval of 2 mm, and the FC17 convolution kernel. The abdominal CT images included in the study did not show any pathological changes involving the coxal bones. All DICOM series were anonymized before their use in the study. The study was approved by the Human Research Ethics Committee at the Institute of Experimental Morphology, Pathology and Anthropology with Museum, Bulgarian Academy of Sciences.

**Figure 1:**
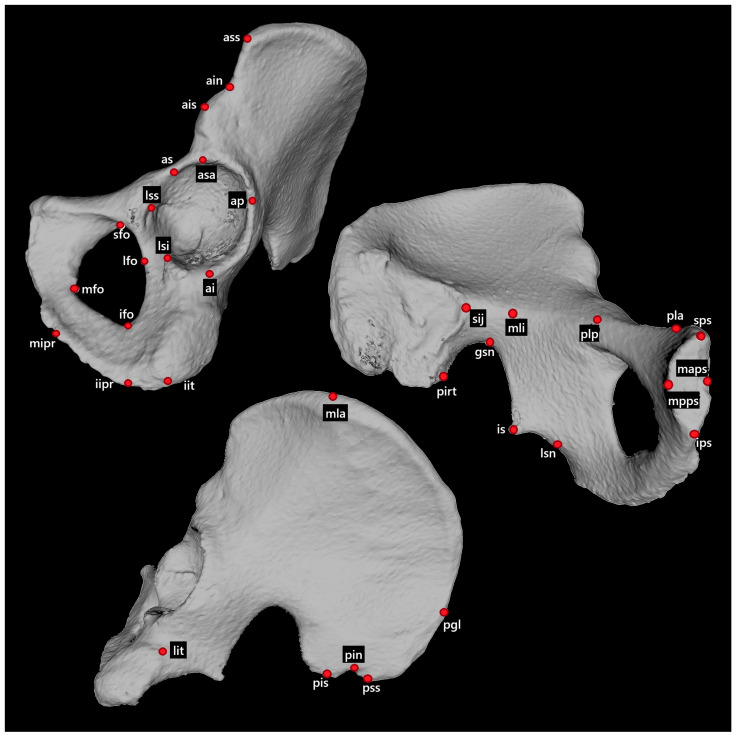
Coxal bone landmarks.

A surface model of the pelvis of each individual was generated from the DICOM series using InVesalius (CTI, São Paulo, Brazil). The segmentation was based on the “Bone” threshold implemented in the software. The 3D models were exported in STL format and used for landmark acquisition. The 3D coordinates of 34 landmarks located on the coxal bone were collected in MeshLab [40] (Figure 1). Out of all 552 left and right coxal bones, 35 bones did not allow the acquisition of the full set of landmarks (one to three missing landmarks), mainly due to the CT scanning process. In most cases, portions of the ischium were not captured within the scanned field, resulting in a not fully reconstructed pelvis. In a few cases, landmarks could not be identified due to surface defects caused by the segmentation process. The missing landmarks were imputed by means of a preprocessing technique for data imputation. In the present study, the Iterative Imputer, using Bayesian Ridge Regression, was employed to fill in the missing coordinates of landmarks. This method considers the relationships between the coordinates of all available landmarks to predict and impute the missing ones. The algorithm works iteratively by cycling through the coordinates with missing values. For each coordinate, it treats the current missing value as a target variable and uses all other available coordinates as predictors. Then, it trains a model using Bayesian Ridge Regression (default in Scikit-learn library [41]) to predict the missing value based on the relationships between the coordinates. This process is repeated for all coordinates with missing values until all gaps are filled.

Based on the landmark coordinates, two datasets of measurements were calculated. The first dataset included mostly standard coxal bone measurements: 27 linear, 3 angular, and 3 height measurements (Table 1), whereas the second dataset consisted of all possible 561 interlandmark distances between the landmarks. The linear measurements were calculated as Euclidean distances. The angles were computed as the angle between two vectors sharing a common origin. The heights were calculated as point-to-line distances, i.e., the orthogonal distance from a landmark to the line connecting two other landmarks, analogous to the height drawn from a vertex onto the opposite side of a triangle.

The coxal bone measurements were tested for significant differences based on sex, age, and bone laterality. To assess the age-related differences between male and female coxal bones, the age of 45 years was selected as a cut-off age (first age group: age ≤ 45 years; second age group: age > 45 years). This age is considered the onset of menopause in women, which is associated with hormonal and metabolic changes that can affect bone structure. The distribution of the data for each group by sex, age and laterality was tested for normality using the Shapiro–Wilk normality test. Depending on the distribution of the data, sex and age groups were compared by the Welch’s *t*-test or the Mann–Whitney U-test, and the right and left measurements were compared by a paired *t*-test or the Wilcoxon signed-rank test. The statistical analyses were conducted using the SciPy library [42].

The ML modeling was performed employing the Scikit-learn library [41]. The models for sex estimation were trained using two algorithms: logistic regression with the “liblinear” solver (with L1 and L2 regularization) and SVM (Support Vector Machine) with the “linear” kernel. The difference between the two types of regularization for logistic regression is that L1 favors sparsity by forcing some coefficients to zero, thereby enabling feature selection, whereas L2 can lead to shrinkage of the coefficients but without eliminating features. The application of a linear kernel in an SVM allows it to operate as a probabilistic method, similar to logistic regression, i.e., to provide the estimated probabilities for each class membership.

Each sex estimation model was trained within a pipeline that applied a StandardScaler to the input features prior to the classifier (SVM or LR). This standardization is especially important when the input features include measurements of varying scales and types to ensure that each feature contributes equally to the model. The optimization of hyperparameter values is another essential step for ensuring good model performance and preventing overfitting or underfitting. In the case of logistic regression, the regularization hyperparameter representing the inverse of the regularization strength was optimized, and thus larger values correspond to weaker regularization, while smaller values imply stronger regularization. For the SVM, the hyperparameter controlling the trade-off between margin width and classification error was optimized. Smaller values of this hyperparameter allow for a wider margin with more tolerance for misclassified points, while larger values result in a narrower margin that fits the training data more closely. Hyperparameter tuning, i.e., the search for optimal parameters, was performed using the Hyperopt-Sklearn library [43]. Optimal hyperparameter values were established based on model performance evaluated via cross-validation within the defined search space (10^−3^ to 10^2^).

Additionally, since L1 regularization in logistic regression enables feature selection, Recursive Feature Elimination with Cross-Validation (RFECV) was applied to select a subset of features for testing the SVM models. RFECV iteratively removes the least important features based on a model’s performance and uses cross-validation to find the optimal number of features that maximize predictive accuracy. This method automatically identifies the most relevant features by optimizing the trade-off between model complexity and accuracy. A Random Forest with 100 trees was employed within the RFECV procedure because Random Forests handle multicollinearity and feature interactions effectively. Using 100 trees ensured stable and reliable feature importance estimates while maintaining a reasonable training time.

The models were trained on right-only, left-only, and combined left-and-right coxal bone data. Each dataset was divided into training and testing sets: 50 bones were allocated for testing in both the right-only and left-only models, while 100 bones were reserved for testing in the combined dataset. The train–test split for ML models trained on either right or left bones was stratified by sex and age. The ‘stratify’ parameter ensured proportional representation of bones from different sexes and age groups in both the training and testing sets. When ML models were developed using both left and right bones together, the train–test split was stratified by sex, age, and laterality. The ML models were trained using 10 × 5-fold cross-validation. For each model, the training accuracy with its confidence interval, as well as the testing accuracy, precision, recall, and F1-score, were reported as percentages. The model performance evaluation was generally based on the testing accuracy, which represents the overall proportion of correct predictions in the sample, and recall values, which provide the proportions of correct predictions within each sex class, analogous to per-class accuracy.

The landmark acquisition in the study was performed by a single experienced observer. The intraobserver error was assessed based on the triple acquisition of the full set of coxal bone landmarks of 30 individuals. Technical errors of measurement (TEMs) and relative technical errors of measurement (rTEMs) for all interlandmark distances were calculated for a quantitative measure of the intraobserver error. The use of the second dataset for testing the error was due to the fact that some of these measurements were included in the first dataset or participated in the calculation of the derivative measurements as angles and heights.

All data processing procedures and analyses were performed with Python 3.11 in JupyterLab [44].

## 3. Results

### 3.1. Intraobserver Error

**Figure 2:**
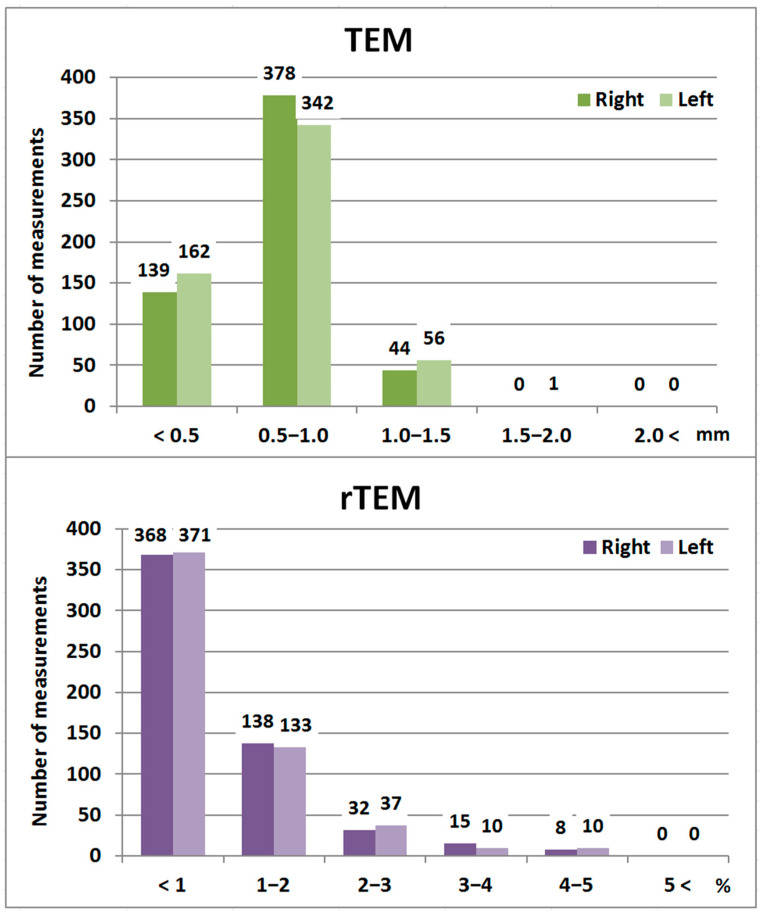
TEM and rTEM values of the coxal bone measurements.

The intraobserver error analysis indicated that the TEMs for all interlandmark distances calculated on the right and left coxal bones (a total of 1122 measurements) were below 2 mm, and all rTEMs were less than 5% (Figure 2, Table S1).

### 3.2. Sex Differences

The sex differences were established for almost all measurements of the first dataset except for the pubic length and transversal diameter of the obturator foramen in both right and left coxal bones, the anterior interspinal distance and pectineal line length in the left bones, and coxal bone breadth in the right bones (Table 2). Four measurements were significantly larger in females than in males: greater sciatic notch breadth, greater sciatic notch angle, ischiopubic ramus length, and posterior interspinal height.

The second dataset showed that significant sex differences were observed in about 85% of the interlandmark distances calculated for the left and right coxal bones (Tables S2–S5).

Only coxal bone measurements demonstrating significant sex differences were used as input variables for the ML models.

### 3.3. Bilateral Differences

Bilateral differences were established in most of the measurements of the first dataset (25 of the measurements in males and 21 of them in females) (Table 3). Most of the significant differences were present in both males and females, and several of them were established only in one of the sexes. The measurements that did not show any significant bilateral differences in the two sexes were as follows: coxal bone height, transversal acetabular diameter, greater sciatic notch breadth, anterior interspinal distance, anterior spino-auricular distance, and pubic symphysis height. In general, the right bones had a wider iliac ala, a longer pubis, a greater vertical acetabular diameter, a greater transversal diameter of the obturator foramen, a longer ischial tuberosity, a greater posterior interspinal distance, a greater spino-sciatic distance, a larger post-acetabular–ischium distance, a wider pubic symphysis, and a deeper greater sciatic notch, while the left coxal bones had a higher iliac ala, a larger gluteo-sciatic distance, a longer ischiopubic ramus, a deeper posterior interspinal notch, a more obtuse ischial angle, and a more obtuse greater sciatic notch angle.

The second dataset showed significant bilateral differences in more than half of the measurements (58% of the male and 56% of the female interlandmark distances) (Table S6).

### 3.4. Age Differences

In males, approximately half of the measurements demonstrated age differences (16 of the left and 15 of the right measurements) (Table 4, Table S7). In females, the results for the left bones were similar, but the right bones showed significant age differences in a lower number of the metrics. Most of the measurements were larger in the older age group. The measurements that showed significant age differences regardless of sex and laterality, i.e., in the right and left bones of the male and female coxal bones, were as follows: iliac breadth at the anterior and posterior superior spines, ischial tuberosity length, greater sciatic–acetabular distance, pubic symphysis height, pubic symphysis width, and anterior interspinal height.

Concerning the second dataset, age differences were observed in about 36% of the right and left interlandmark distances of the male coxal bones. The female bones demonstrated again a slightly lower proportion of observed significant age differences: in 35% of the total number of measurements characterizing the left coxal bones and in 31% of those describing the right coxal bones (Table S8).

### 3.5. ML Models

#### 3.5.1. Logistic Regression

**Figure 3:**
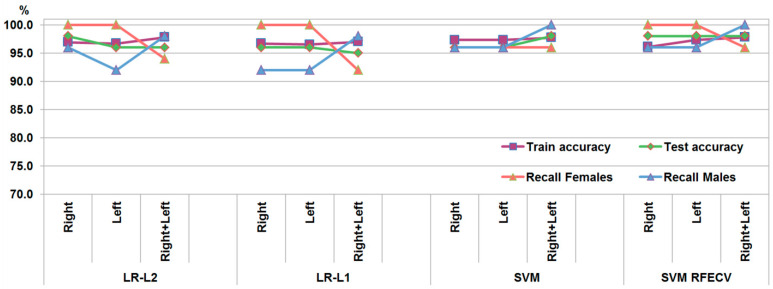
Performance metrics of the models trained on the first dataset.

LR models with L2 regularization (LR-L2) trained on the first dataset provided accuracy rates of 98% for the right bones, 96% for the left bones, and 96% for the combined right and left bones (Figure 3). The use of L1 regularization (LR-L1) with a reduction in the measurements provided an accuracy of 96% for the right and left bones separately and an accuracy of 95% for the combined left and right bones. After applying L1 regularization, the number of measurements with non-zero coefficients was reduced to 22 for the left coxal bones, 24 for the right coxal bones, and 27 for the model trained on combined left and right bones (Table S9). Most of the measurements included in the three LR-L1 models were common; among them were the features characterizing the greater sciatic notch, coxal bone height and iliac breadth at superior spines, pubic symphysis width, vertical acetabular diameter, the vertical diameter of the obturator foramen, intersciatic distance, and interspinal distances.

**Figure 4:**
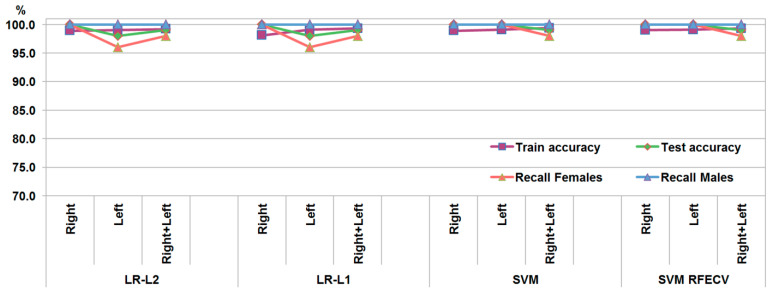
Performance metrics of the models trained on the second dataset.

The second dataset provided identical accuracy rates for the LR models with L1 and L2 regularization: 98% for the left bones, 100% for the right bones, and 99% for the combined left and right bones (Figure 4). The LR-L1 model kept 83 interlandmark distances with non-zero coefficients for the left bones, 63 distances for the right bones, and 28 measurements for the combined left and right bones (Table S9). Out of these measurements, 32 distances were common for the left and right bones, but only 15 of them were present in the LR-L1 model trained on the combined dataset of the right and left bones. Among the selected features, most were between landmarks located on the iliac ala (mla, asa, and pis), the foramen obturatum margin (mfo, ifo, sfo, and lfo), and the pubic symphysis (mpps and ips).

#### 3.5.2. SVM

The SVM models trained on the first dataset provided accuracy rates of 96% for the separate right and left measurements and 98% for the combined right and left bones (Figure 3). After selecting the features contributing the most to the sex discrimination by RFECV, the number of features was reduced to 27 for the left bones, 18 for the right bones, and 22 for the combined left and right bones (Table S9). Most of the features selected in the three subsets coincided with those kept in the LR-L1 models (pubic symphysis width, vertical acetabular diameter, vertical diameter of the obturator foramen, ischiopubic angle, coxal bone height, and intersciatic distance). Training SVM models on these subsets resulted in equal accuracy (98%) for the left-only, right-only, and combined left and right bones.

The SVM models trained on the measurements of the second dataset achieved accuracy rates of 100% for the separate right and left bones and 99% for the combined right and left bones (Figure 4). After selecting the features with RFECV, the same accuracy rates were achieved, although the number of features was reduced considerably to 60 measurements for the left bones, 25 measurements for the right bones, and 137 measurements for the subset combining right and left bones (Table S9). Twenty-four of the measurements were common for the three subsets. So, these measurements were the ones strongly related to sex and mostly represented distances constructed between landmarks located on the pubic symphysis (maps, mpps, sps, and ips), the ischiopubic ramus (mipr and iipr), the pectineal line (pla and plp), the foramen obturatum (lfo, ifo, and sfo), and the acetabulum (asa and as), i.e., placed in the anterior part of the bone. However, these distances did not correspond to any of those from the first dataset and connected the separate coxal bone structures instead of characterizing them individually.

All ML models provided close values for training and testing accuracy (Table S10). Overall, the ML models trained on the second dataset yielded higher accuracy rates and showed less sex bias. In addition, the SVM models provided more consistent evaluation metrics than the LR models. Feature selection generally produced different results only for the first dataset, with slightly worse performance using L1 regularization and slightly better performance using RFECV. For the second dataset, feature selection yielded similar results before and after the procedure, thus achieving the same result with a reduced number of features.

All trained models were saved in .pkl format and are provided in File S11. They can be reused by loading them into a Python environment using the pickle library. This allows inspection of the features used in the models as well as their hyperparameter settings. The models can be applied to new cases, provided the input measurements match those used for training, allowing the prediction of class probabilities.

## 4. Discussion

Sexual dimorphism in pelvic bones has been examined in many works, particularly in the field of forensic anthropology. Numerous studies have established significant sex differences in coxal bone dimensions [16,21,22,25,28,45]. Several studies have found sex differences not in specific measurements but in the centroid size of male and female coxal bones [29,30,46]. Our results demonstrated that the majority of coxal bone measurements differed significantly between males and females, in line with the previous findings. However, a few of the measurements did not show significant sex differences, which has also been reported in previous studies for pubic length [5,16,21,28,45], coxal breadth [21,28], and the transversal diameter of the obturator foramen [25,28]. In addition, non-significant sex differences were observed for the left-side anterior interspinal distance and the pectineal line length, but these were not reported in earlier studies. In the present study, male coxal bones were generally larger in size, with only four measurements found to be greater in females. Two of these measurements were related to the greater sciatic notch, its breadth and angle, and so far they have been established as being larger in females [21,26]. It has been deduced that the overall size of the coxal bone reflects the pattern of body size sexual dimorphism, with males being larger than females [46]. In particular, greater sex differences in coxal bone measurements have been observed along the vertical axis of the body, especially in coxal bone height [21]. Although the coxal bones themselves are larger in males, it has been established that the pelvic cavity is more spacious in females [5], which is attributed to the shape and size differences in male and female coxal bones. According to Betti [21], the larger pelvic cavities in females are primarily due to differences in the shape of the coxal bones rather than explicit size differences. However, the present study provided metric data about the greater sciatic notch and the ischiopubic region that can be associated with the spaciousness of the female pelvic canal.

The few studies that have developed ML classification models based on pelvic characteristics have used different algorithms and reported varying levels of accuracy. Coelho and Curate [38] trained twelve ML algorithms for sex estimation using pelvic metric variables, with the highest accuracy of 97.3% achieved by LR with L2 regularization, a neural network, and partial least squares. Similarly, Secgin et al. [33] constructed sex estimation models using five classifiers, with the best performance achieved by DA (96%), followed by the ADABoost classifier (94%) and LR, which reached up to 92% accuracy. Both studies used metric characteristics but did not apply SVMs in their experiments. However, previous ML models based on cranial and mandibular measurements have identified SVMs as the best-performing algorithms, closely followed by LR [47,48,49]; therefore, the present study focused on training SVM and LR algorithms and considered their ability to provide class probabilities. Furthermore, Nikita and Nikitas [23] developed ML models using scores for pelvic traits (the ventral arc, the subpubic concavity, and the medial aspect of the ischiopubic ramus) and achieved accuracy rates of around 95% with artificial neural networks, DA, and naïve Bayes, but only 91.5% with LR. Other studies have employed DL to evaluate the classification potential of features derived from images of the coxal bones and the pelvis as a whole. Cao et al. [34] used a convolutional neural networkto classify male and female pelves based on images of six anatomical regions; four of them (the ventral pubis, the dorsal pubis, the greater sciatic notch, and the pelvis inlet) achieved accuracy rates of 95% or higher, while the ischium and acetabulum yielded lower accuracy values (below 83%). Epain et al. [35] reported very high classification performance, achieving accuracy rates of 97.9% and 99.8% by training two variational autoencoders on 3D meshes of paired right and left coxal bones. The accuracy results obtained in the present study (95–100%) were generally higher than those of most previously published results. The comparable accuracy observed for both the training and testing datasets indicates that the models are neither overfitting nor underfitting. Moreover, combining the datasets of right and left bones did not result in overfitting, which might have been expected given the symmetry between the right and left coxal bones within individuals in the sample. Therefore, despite the limited sample size in the present study, the models converged well and performed effectively on previously unseen data.

The results of all the aforementioned ML and DL studies demonstrate accuracy rates above 95% for at least one or a few trained models, with performance comparable to or exceeding that of classical studies based solely on DA. The Diagnose Sexuelle Probabiliste (DSP) method [12] and DSP2 software [39], developed based on coxal bone measurements derived from different population groups, successfully classified male and female bones with 99–100% accuracy. Subsequent studies verifying these methods reported accuracy rates ranging from 85.7% to 100% [22,45,50]. In other studies that have developed discriminant functions for particular populations, the best accuracy rates varied in almost the same range: 87.6–94.8% in Greeks [16,22], 94.3–100% in Americans [6,32], 96.7% in a Japanese sample [6], 94.7% in the Spanish population [21], 94.5% in South Africans [16], and 89.7% in the Thai population [25]. Almost all of the best discriminant functions included coxal bone height, iliac width, acetabular diameters, and greater sciatic notch variables as input variables. According to Kimura [6], among the dimensions of the three bones forming the coxal bone, the metric characteristics of the pubic bone are the most important for sexual dimorphism. Consistent with this, Luo [9] achieved 100% accuracy using discriminant functions based on only four pubic measurements. Similarly, Baca et al. [31] applied a geometric morphometric (GM) approach with eight landmarks characterizing the pubic bone and achieved an accuracy of 95.5%. In the present study, the pubic measurements selected after feature selection were pubic symphysis width and ischiopubic angle from the first dataset, while landmarks describing the pubic symphysis contributed to many interlandmark distances selected from the second dataset by RFECV. Thus, pubic-related measurements contributed to sex discrimination; however, it is important to note that pubic length did not differ significantly between the sexes and was not included in any of the models. The absence of sex differences in this measurement indicates that the pubic bone length is nearly the same in both females and males, despite males having generally larger bones. This allows for a proportionally enlarged pelvic inlet in females, serving as an adaptation to facilitate parturition [29], along with the wider greater sciatic notch and the longer and more oblique ischiopubic ramus, contributing to a more spacious pelvic canal inferiorly.

Most previous studies that examined asymmetry in the coxal bone found no bilateral differences in the measured parameters [21] or only in a few of them, such as cotylo-pubic breadth [28]; ischium length, pubic length, and acetabular height [51]; and iliac breadth and pubic length [52]. Moreover, Decker et al. [32] established strong correlations between complementary bilateral measurements, with negligible variation between the right and left coxal bones. However, we found sex differences in more than half of the studied measurements, indicating the presence of pronounced bilateral asymmetry in the coxal bones of both males and females. The bilateral differences revealed that the iliac ala is wider on the right and higher on the left side; the greater sciatic notch is deeper on the right and wider on the left side; and the pubic bone is longer and has a wider symphysis on the right, whereas the ischiopubic ramus is longer on the left side. Kurki [52] reported significant bilateral differences only in the coxal bone measurements of males, with pubic bone length being left-biased and iliac breadth right-biased. However, in the present study, these two measurements showed significant right-sided asymmetry in both males and females. The differences between previous studies and the present findings demonstrate the need for further examination of coxal bone asymmetry. The presence of significant differences in more than half of the studied measurements suggests that the right and left coxal bones differ considerably in their metric characteristics, and researchers should be cautious about using left and right bones interchangeably. However, the pattern of asymmetry with respect to side bias is generally similar in both males and females. To assess whether laterality affects classification accuracy, we trained sex estimation models on combined data from both left and right coxal bones. It is important to note that combining left and right bones in a single dataset is valid, as the presence of bilateral differences ensures that they are not identical, eliminating the risk of duplicate bones appearing in both the training and testing sets. In addition, combining data from both left and right bones allowed for an increased sample size (doubling it), which is particularly important for developing ML models. Stratifying the train–test split ensures equal representation of right and left bones in both the training and testing sets, preventing overrepresentation of one side in the test sample. It is important to note that, in practice, both right and left coxal bones are not always available for examination. Therefore, models developed using combined data from both sides are valuable, as they learn from coxal bone measurements derived from both the left and right sides. The similar performance of the models on right-only, left-only, and combined data suggests that sexual dimorphism in the coxal bone is strong and that the differences between males and females outweigh the side-related variation. Hence, the models were able to separate the sexes effectively, despite some intrasex variation due to side. Consistent with the results of the present study, Baca et al. [31] demonstrated that symmetry between the right and left coxal bones does not substantially bias the sample toward higher predictive power of developed models.

Age-related differences in coxal bone features have been examined in a few studies, but they have focused on specific regions rather than the entire bone. Anastasiou et al. [18] found that the individual’s age has no significant effect on the size of the auricular surfaces. Similarly, age does not significantly influence the shape of either the greater sciatic notch or the obturator foramen [24]. On the other hand, Walker [13] established a strong correlation between sciatic notch scores and age at death for individuals under 50, while for older individuals this relationship was not statistically significant. In the present study, age-related differences were found in approximately one-third of the measurements. All significant distances were in favor of the older individuals and were mainly related to measurements including iliac spines, pubic symphysis, ischial tuberosity, as well as acetabular diameters and the vertical diameter of the obturator foramen. These results could be related to bone changes (deposition or resorption) occurring in certain areas (e.g., joints and bone spines) with advancing age, primarily as a consequence of age-related metabolic changes. It has been established that the female pelvis reaches its most obstetrically adequate morphology around the peak of fertility (25–30 years), but around the age of 40–45 years, it reverts to a developmental pattern similar to that of males, accompanied by a significant reduction in the dimensions of the birth canal [53]. These changes in the female pelvis during the postmenopausal period could be linked to a decrease in steroid hormone secretion [54]. However, the age differences identified in the present study involved a slightly smaller number of coxal bone measurements in females compared to males, indicating that active age-related changes occur in the male pelvis as well.

Sex estimation in forensic and biological anthropology becomes more complex due to population-specific variation [27]. According to Patriquin et al. [23], population differences influence the expression of sexual dimorphism in pelvic bones and must be considered when developing the most effective sex estimation methods. Kimura [6] established population differences in coxal bone measurements within each sex, and Sorrentine et al. [28] even found regional variations in hip bone metrics within the Italian population. However, some previous studies have supported the hypothesis that pelvic sexual variation is not structured in a population-specific manner [12,16]. Steyn and Patriquin [16] have suggested that pelvic dimensions are constrained by the demands of childbirth to such an extent that interpopulation differences in these dimensions are negligible. In support of this, Fisher et al. [55] stated that all populations exhibit a very similar pattern of pelvic sexual dimorphism despite wide variation in the magnitude of observed sex differences. Hence, the discrepancies in observed accuracy rates across the studies could be explained not only by the choice of coxal bone features but also by the source material, derived from different geographic and temporal populations.

The present results show that the Bulgarian population exhibits strong sexual dimorphism in coxal bone features. The accuracy rates achieved exceed those of ML models based on cranial and mandibular measurements derived from the same population [47,48,49]. This confirms ones again the leading role of the pelvis in the sex estimation process [56,57,58]. The present study introduces a methodology for sex estimation that differs from traditional approaches, which mainly rely on DA applied to a limited set of direct bone measurements. The proposed workflow involves collecting 3D coordinates from 34 anatomical landmarks and computing numerous derived measurements, which is time-consuming but yields an enriched dataset that enhances model accuracy on the tested sample. However, recent advancements in automated landmarking tools have the potential to significantly reduce this effort. A limitation of the developed ML models is their reliance on intact coxal bones, which may hinder their applicability in cases involving incomplete bones. Nevertheless, missing landmarks can be imputed using appropriate algorithms when a sufficiently large dataset of landmark data is available. Furthermore, although commonly used pelvic sex estimation tools have not been validated on the Bulgarian dataset, the feature selection procedures applied in this study indicate that the best classification results are achieved with larger variable sets compared to the more limited measurement sets involved in previous models. While this divergence from former methods introduces additional complexity, it enables the exploration of alternative approaches that incorporate diverse measurement sets and advanced algorithms. So far, the ML models developed in this study provide the highest accuracy for sex estimation based on coxal bone data derived from a Bulgarian sample.

## 5. Conclusions

Coxal bone measurements have proved to be valuable features for sex estimation, providing successful classification of the male and female coxal bones. SVM and LR models effectively estimate sex using both full datasets and subsets of selected coxal bone features. The accuracy of the provided models ranges between 95% and 100%. The highest accuracy was achieved by a combination of interlandmark distances connecting landmarks from different structures of the coxal bone, such as the pubic symphysis, foramen obturatum, and acetabulum, rather than characterizing them as entities. Despite being trained on coxal bone measurements potentially affected by bilateral and age-related differences, the ML models yielded high accuracy rates, suggesting that such variation did not hinder sex estimation.

## Figures and Tables

**Table 1:** Coxal bone measurements included in the first dataset.

Measurement	Description
Coxal bone height (mla-iit)	The linear distance from the most lateral point of the iliac ala (mla) to the most inferior point of the ischial tuberosity (iit).
Coxal bone breadth (sps-pss)	The linear distance from the superior point of the pubic symphysis (sps) to the posterior superior spine (pss).
Ala breadth at superior iliac spines (ass-pss)	The linear distance from the anterior superior spine (ass) to the posterior superior spine (pss).
Ala breadth at inferior iliac spines (ais-pis)	The linear distance from the anterior inferior spine (ais) to the posterior inferior spine (pis).
Ala height (mla-mli)	The linear distance from the most lateral point of the iliac ala (mla) to the most lateral point of the pelvic inlet (mli).
Pubic length (sps-as)	The linear distance from the superior point of the pubic symphysis (sps) to the superior point of the acetabulum at the pubic–iliac junction (as).
Vertical acetabular diameter (asa-ai)	The linear distance from the superior point of the acetabular margin at the base of iliac ala (asa) to the inferior point of the acetabular margin (ai).
Transversal acetabular diameter (ap-lss)	The linear distance from the posterior point of the acetabular margin (ap) to the most medial point of the superior portion of the lunate surface (lss).
Vertical diameter of obturator foramen (sfo-ifo)	The linear distance from the most superior (sfo) to the most inferior (ifo) point of the margin of the obturator foramen.
Transversal diameter of obturator foramen (mfo-lfo)	The linear distance from the most medial (mfo) to the most lateral (lfo) point of the obturator foramen.
Greater sciatic notch breadth (pirt-is)	The linear distance from the piriform tubercle (pirt) to the ischial spine (is).
Ischiopubic ramus length (ips-iipr)	The linear distance from the inferior point of the pubic symphysis (ips) to the inferior point of the ischiopubic ramus (iipr).
Ischial tuberosity length (lit-iipr)	The linear distance from the most lateral point of the ischial tuberosity (lit) to the inferior point of the ischiopubic ramus (iipr).
Intersciatic distance (gsn-lsn)	The linear distance between the deepest points of greater (gsn) and lesser (lsn) sciatic notches.
Anterior interspinal distance (ass-ais)	The linear distance from the anterior superior spine (ass) to the anterior inferior spine (ais).
Posterior interspinal distance (pss-pis)	The linear distance from the posterior superior spine (pss) to the posterior inferior spine (pis).
Spino-sciatic distance (ais-gsn)	The linear distance from the anterior inferior spine (ais) to the deepest point of the greater sciatic notch (gsn).
Anterior spino-auricular distance (ais-sij)	The linear distance from the anterior inferior spine (ais) to the most anterior point of the sacroiliac joint on the outline of the pelvic inlet (sij).
Posterior spino-auricular distance (pis-sij)	The linear distance from the posterior inferior spine (pis) to the most anterior point of the sacroiliac joint (sij).
Post-acetabular–ischium distance (ap-iipr)	The linear distance from the posterior point of the acetabular margin (ap) to the inferior point of the ischiopubic ramus (iipr).
Gluteo-sciatic distance (pgl-gsn)	The linear distance from the crossing point of the iliac crest and the posterior gluteal line (pgl) to the deepest point of the greater sciatic notch (gsn).
Pectineal line length (pla-plp)	The linear distance from the anterior (pla) to the posterior (plp) end of the pectineal line.
Lunate–ramus distance (mipr-lsi)	The linear distance from the mid-point of the ischiopubic ramus (mipr) to the most medial point of the inferior portion of the lunate surface (lsi).
Greater sciatic–acetabular distance (gsn-ap)	The linear distance from the deepest point of the greater sciatic notch (gsn) to the most posterior point of the acetabular margin (ap).
Lesser sciatic–acetabular distance (lsn-ai)	The linear distance from the deepest point of the lesser sciatic notch (lsn) to the inferior point of the acetabular margin (ai).
Pubic symphysis height (sps-ips)	The linear distance from the superior (sps) to the inferior (ips) point of the pubic symphysis.
Pubic symphysis width (maps-mpps)	The linear distance between the mid-anterior (maps) and mid-posterior (mpps) points of the pubic symphysis.
Greater sciatic notch height (gsn_pirt-is)	The projection distance from the deepest point of the greater sciatic notch (gsn) to the line defined by the piriform tubercle (pirt) and ischial spine (is).
Anterior interspinal height (ain_ass-ais)	The projection distance from the deepest point of the anterior interspinous notch (ain) to the line defined by the anterior superior spine (ass) and anterior inferior spine (ais).
Posterior interspinal height (pin_pss-pis)	The projection distance from the deepest point of the posterior interspinous notch (pin) to the line defined by the posterior superior spine (pss) and posterior inferior spine (pis).
Greater sciatic notch angle (pirt-gsn-is)	The angle formed between the lines pirt-gsn and gsn-is, with the vertex at the deepest point of the greater sciatic notch (gsn).
Ischiopubic angle (sps-ips-iipr)	The angle formed between the lines sps-ips and ips-iipr, with the vertex at the inferior point of the pubic symphysis (ips).
Ischial angle (mipr-iit-lit)	The angle formed between the lines mipr-iit and iit-lit, with the vertex at the most inferior point of the ischial tuberosity (iit).

**Table 2:** Descriptive statistics and significance of sex differences in measurements of the first dataset.

Measurements	Scheme	Males				Females				Sex. Diff. *p*-Value
		Mean	STD	Min	Max	Mean	STD	Min	Max	
Coxal bone height	Right	210.55	9.93	190.32	236.83	190.49	9.16	170.13	224.80	<0.001 ^U^
	Left	210.06	9.61	191.13	236.08	190.11	9.31	167.82	222.64	<0.001 ^t^
Coxal bone breadth	Right	178.90	12.55	144.64	213.02	177.13	10.99	147.43	205.69	0.213 ^t^
	Left	178.02	12.22	144.57	211.65	175.04	10.97	149.95	198.92	0.034 ^t^
Ala breadth at superior iliac spines	Right	165.80	8.71	147.96	183.26	159.95	8.02	140.89	180.78	<0.001 ^t^
	Left	165.12	8.87	146.12	184.27	159.18	7.82	137.13	177.29	<0.001 ^t^
Ala breadth at inferior iliac spines	Right	124.57	8.58	106.31	143.26	120.50	8.27	102.39	141.65	<0.001 ^t^
	Left	125.42	7.54	108.36	145.23	120.63	8.28	99.40	140.04	<0.001 ^t^
Ala height	Right	105.07	5.79	92.05	123.26	98.41	4.69	86.90	114.61	<0.001 ^t^
	Left	106.33	6.05	92.17	125.42	98.85	4.59	89.37	110.36	<0.001 ^t^
Pubic length	Right	84.04	5.84	64.98	97.24	84.00	5.31	71.81	99.54	0.957 ^t^
	Left	83.21	5.98	68.03	99.71	83.36	5.42	71.08	99.91	0.832 ^t^
Vertical acetabular diameter	Right	57.88	3.59	50.74	69.83	52.10	3.03	43.22	59.68	<0.001 ^U^
	Left	57.17	3.53	48.45	66.98	51.50	2.79	44.73	59.59	<0.001 ^t^
Transversal acetabular diameter	Right	56.55	3.51	48.10	67.07	50.34	3.09	43.50	57.77	<0.001 ^U^
	Left	56.25	3.64	45.16	66.72	50.19	3.22	40.39	60.98	<0.001 ^t^
Vertical diameter of obturator foramen	Right	57.87	4.66	48.38	74.63	52.19	3.68	43.14	61.36	<0.001 ^U^
	Left	57.60	4.55	48.61	69.72	52.53	3.80	42.97	65.05	<0.001 ^t^
Transversal diameter of obturator foramen	Right	35.94	3.51	24.40	48.44	35.82	2.66	30.60	42.82	0.670 ^U^
	Left	35.51	3.50	26.85	49.21	35.55	2.93	29.92	44.75	0.832 ^U^
Greater sciatic notch breadth	Right	51.53	5.34	39.65	65.80	54.72	6.25	40.55	72.45	<0.001 ^t^
	Left	51.28	5.38	40.21	64.99	54.23	5.95	41.98	68.30	<0.001 ^U^
Ischiopubic ramus length	Right	58.73	5.41	44.52	74.99	61.14	5.77	49.63	81.32	<0.001 ^t^
	Left	62.21	5.91	44.21	76.69	64.00	5.54	51.17	82.14	0.010 ^t^
Ischial tuberosity length	Right	68.39	4.98	57.11	86.69	62.50	4.82	52.52	75.62	<0.001 ^t^
	Left	67.15	4.94	50.56	81.80	61.49	4.61	50.41	73.23	<0.001 ^t^
Intersciatic distance	Right	61.30	5.60	48.34	76.84	55.48	4.51	41.57	68.68	<0.001 ^t^
	Left	60.50	5.17	46.96	71.90	55.04	4.40	43.70	69.34	<0.001 ^t^
Anterior interspinal distance	Right	43.52	5.92	23.21	59.66	42.21	5.05	27.56	54.29	0.050 ^t^
	Left	43.09	5.89	23.93	57.88	42.87	5.48	25.87	55.80	0.744 ^t^
Posterior interspinal distance	Right	39.52	9.34	19.14	63.48	33.45	7.93	16.62	53.52	<0.001 ^U^
	Left	37.79	8.46	17.70	62.51	31.94	7.51	15.17	50.85	<0.001 ^U^
Spino-sciatic distance	Right	82.41	4.94	70.51	98.35	74.14	4.64	63.76	84.56	<0.001 ^t^
	Left	81.76	4.97	69.92	96.44	73.71	4.69	63.70	83.83	<0.001 ^t^
Anterior spino-auricular distance	Right	81.34	5.43	67.13	93.98	79.65	5.82	64.67	92.30	0.013 ^t^
	Left	81.71	6.50	67.87	107.48	79.96	5.59	64.82	96.52	0.041 ^U^
Posterior spino-auricular distance	Right	55.35	6.14	41.61	69.94	49.08	6.15	33.56	64.13	<0.001 ^t^
	Left	54.14	5.89	37.03	69.41	48.78	6.32	31.55	65.09	<0.001 ^t^
Post-acetabular–ischium distance	Right	98.24	6.73	85.12	120.92	86.32	5.31	74.73	100.45	<0.001 ^U^
	Left	96.24	6.42	81.24	114.91	85.39	5.95	69.92	101.88	<0.001 ^t^
Gluteo-sciatic distance	Right	94.53	5.44	80.90	109.31	91.97	4.62	83.47	107.13	<0.001 ^U^
	Left	95.63	5.59	81.52	115.81	93.18	4.79	79.46	105.08	<0.001 ^t^
Pectineal line length	Right	33.31	6.11	15.69	50.92	31.02	5.67	16.18	53.37	<0.001 ^U^
	Left	30.68	6.39	14.80	44.64	30.41	5.94	16.14	50.13	0.719 ^t^
Lunate–ramus distance	Right	61.81	4.38	49.65	72.29	56.19	3.69	46.63	67.41	<0.001 ^t^
	Left	61.15	4.72	49.30	76.08	56.02	3.70	45.80	64.80	<0.001 ^t^
Greater sciatic–acetabular distance	Right	49.64	4.16	40.39	64.07	46.17	4.07	36.56	56.94	<0.001 ^t^
	Left	50.29	4.64	40.12	67.70	46.73	4.18	37.92	57.54	<0.001 ^U^
Lesser sciatic–acetabular distance	Right	41.97	4.39	21.56	55.17	37.96	3.14	30.93	49.77	<0.001 ^U^
	Left	43.11	3.58	34.06	56.23	38.58	2.85	32.40	50.99	<0.001 ^U^
Pubic symphysis height	Right	39.94	4.92	26.83	52.89	35.76	3.69	26.86	44.33	<0.001 ^t^
	Left	39.78	4.60	26.68	52.07	35.77	3.88	26.63	44.61	<0.001 ^t^
Pubic symphysis width	Right	18.40	2.46	12.49	27.40	15.07	2.19	10.15	21.65	<0.001 ^U^
	Left	18.08	2.47	11.56	26.25	14.79	2.34	8.59	21.72	<0.001 ^t^
Greater sciatic notch height	Right	30.10	3.71	22.07	38.34	29.10	3.45	19.42	38.00	0.021 ^t^
	Left	29.36	3.51	19.57	38.25	28.25	3.09	21.48	35.70	0.006 ^t^
Anterior interspinal height	Right	8.66	2.10	3.95	17.45	7.55	1.29	4.70	12.07	<0.001 ^U^
	Left	8.26	1.98	3.63	16.42	7.52	1.47	4.62	11.70	<0.001 ^U^
Posterior interspinal height	Right	3.71	1.80	0.46	9.21	4.71	2.85	0.53	15.99	0.005 ^U^
	Left	4.51	2.63	0.81	16.32	5.17	2.62	0.35	14.63	0.014 ^U^
Greater sciatic notch angle	Right	68.61	6.55	50.29	85.93	80.51	7.97	59.59	101.83	<0.001 ^t^
	Left	70.22	6.77	49.76	85.19	81.91	7.60	60.53	100.36	<0.001 ^t^
Ischiopubic angle	Right	142.65	4.46	129.78	154.57	132.64	4.12	117.74	143.30	<0.001 ^t^
	Left	142.85	4.62	130.63	156.49	133.28	4.09	123.01	148.16	<0.001 ^t^
Ischial angle	Right	99.09	4.23	85.80	110.86	97.11	4.33	87.29	109.15	<0.001 ^t^
	Left	100.92	4.91	87.91	114.59	98.74	4.33	87.39	112.57	<0.001 ^t^

t—Welch’s *t*-test; U—Mann–Whitney U-test.

**Table 3:** Bilateral differences in the measurements from the first dataset.

Measurements	Males		Females	
	Abs. Difference *	*p*-Value	Abs. Difference	*p*-Value
Coxal bone height	0.49	0.075 ^t^	0.37	0.166 ^W^
Coxal bone breadth	0.88	0.025 ^t^	2.08	<0.001 ^t^
Ala breadth at superior iliac spines	0.68	0.013 ^t^	0.76	0.003 ^t^
Ala breadth at inferior iliac spines	−0.85	0.049 ^t^	−0.13	0.774 ^t^
Ala height	−1.26	<0.001 ^t^	−0.45	0.048 ^t^
Pubic length	0.83	0.010 ^t^	0.65	0.026 ^t^
Vertical acetabular diameter	0.72	<0.001 ^W^	0.60	0.001 ^t^
Transversal acetabular diameter	0.30	0.070 ^W^	0.15	0.446 ^t^
Vertical diameter of obturator foramen	0.28	0.211 ^W^	−0.34	0.033 ^t^
Transversal diameter of obturator foramen	0.42	0.006 ^t^	0.27	0.029 ^W^
Greater sciatic notch breadth	0.24	0.394 ^t^	0.49	0.125 ^t^
Ischiopubic ramus length	−3.47	<0.001 ^t^	−2.85	<0.001 ^t^
Ischial tuberosity length	1.23	<0.001 ^t^	1.01	<0.001 ^t^
Intersciatic distance	0.80	0.005 ^t^	0.43	0.057 ^t^
Anterior interspinal distance	0.43	0.331 ^t^	−0.66	0.060 ^t^
Posterior interspinal distance	1.72	0.001 ^W^	1.50	0.005 ^W^
Spino-sciatic distance	0.65	0.009 ^t^	0.42	0.007 ^t^
Anterior spino-auricular distance	−0.36	0.134 ^W^	−0.31	0.333 ^t^
Posterior spino-auricular distance	1.21	0.002 ^t^	0.30	0.525 ^t^
Post-acetabular–ischium distance	2.00	<0.001 ^W^	0.93	0.013 ^t^
Gluteo-sciatic distance	−1.11	<0.001 ^t^	−1.21	<0.001 ^W^
Pectineal line length	2.63	<0.001 ^t^	0.61	0.256 ^W^
Lunate–ramus distance	0.66	0.004 ^t^	0.17	0.303 ^t^
Greater sciatic–acetabular distance	−0.65	0.022 ^W^	−0.56	0.029 ^W^
Lesser sciatic–acetabular distance	−1.14	<0.001 ^W^	−0.61	<0.001 ^W^
Pubic symphysis height	0.15	0.415 ^t^	−0.01	0.937 ^t^
Pubic symphysis width	0.32	0.001 ^t^	0.28	0.044 ^W^
Greater sciatic notch height	0.75	0.004 ^t^	0.85	<0.001 ^t^
Anterior interspinal height	0.41	0.006 ^W^	0.03	0.725 ^W^
Posterior interspinal height	−0.81	0.001 ^W^	−0.46	0.001 ^W^
Greater sciatic notch angle	−1.61	<0.001 ^t^	−1.40	0.002 ^t^
Ischiopubic angle	−0.20	0.470 ^t^	−0.64	0.026 ^t^
Ischial angle	−1.84	<0.001 ^t^	−1.63	<0.001 ^t^

*—(right measurement–left measurement); t—paired *t*-test; W—Wilcoxon signed-rank test.

**Table 4:** Age differences in the measurements from the first dataset.

Measurements	Males				Females			
	Right		Left		Right		Left	
	Abs. Diff. *	*p*-Value	Abs. Diff.	*p*-Value	Abs. Diff.	*p*-Value	Abs. Diff.	*p*-Value
Coxal bone height	3.35	0.114 ^t^	2.31	0.265 ^t^	3.31	0.165 ^U^	4.41	0.040 ^U^
Coxal bone breadth	4.44	0.900 ^t^	5.38	0.041 ^t^	5.27	0.037 ^t^	5.54	0.024 ^t^
Ala breadth at superior iliac spines	6.04	<0.001 ^t^	5.67	0.001 ^t^	4.49	0.010 ^t^	5.38	0.003 ^t^
Ala breadth at inferior iliac spines	2.15	0.168 ^t^	3.19	0.024 ^t^	1.54	0.410 ^t^	2.61	0.177 ^t^
Ala height	3.01	0.014 ^t^	2.86	0.024 ^t^	1.14	0.329 ^t^	1.80	0.064 ^t^
Pubic length	3.34	0.006 ^t^	4.12	<0.001 ^t^	1.11	0.374 ^t^	0.89	0.442 ^t^
Vertical acetabular diameter	1.42	0.040 ^U^	0.88	0.182 ^t^	1.04	0.125 ^t^	0.97	0.150 ^t^
Transversal acetabular diameter	2.33	<0.001 ^t^	1.88	0.014 ^t^	1.05	0.095 ^t^	1.34	0.018 ^U^
Vertical diameter of obturator foramen	2.04	0.009 ^U^	2.42	0.009 ^t^	1.69	0.036 ^t^	1.72	0.053 ^t^
Transversal diameter of obturator foramen	0.08	0.912 ^t^	−0.21	0.779 ^t^	1.09	0.120 ^U^	1.22	0.090 ^U^
Greater sciatic notch breadth	−0.04	0.969 ^t^	−0.89	0.351 ^t^	2.65	0.041 ^t^	2.40	0.065 ^t^
Ischiopubic ramus length	−0.36	0.714 ^t^	0.18	0.865 ^t^	−0.30	0.793 ^t^	−0.83	0.469 ^t^
Ischial tuberosity length	2.90	0.003 ^t^	2.66	0.004 ^t^	3.00	0.004 ^t^	3.74	<0.001 ^t^
Intersciatic distance	1.62	0.134 ^t^	0.22	0.799^U^	1.60	0.179 ^t^	1.98	0.058 ^t^
Anterior interspinal distance	0.41	0.698 ^t^	−0.11	0.913 ^t^	0.70	0.583 ^t^	1.20	0.285 ^t^
Posterior interspinal distance	3.71	0.034 ^t^	3.56	0.076 ^U^	4.52	0.003 ^t^	3.31	0.042 ^U^
Spino-sciatic distance	3.18	<0.001 ^t^	2.62	0.005 ^t^	1.53	0.169 ^t^	1.57	0.164 ^t^
Anterior spino-auricular distance	0.40	0.705 ^t^	0.58	0.692 ^U^	−0.43	0.761 ^t^	−0.12	0.930 ^t^
Posterior spino-auricular distance	2.23	0.045 ^U^	2.42	0.027 ^U^	0.62	0.883 ^U^	1.53	0.253 ^t^
Post-acetabular–ischium distance	3.25	0.011 ^U^	2.73	0.016 ^t^	1.07	0.372 ^t^	3.49	0.003 ^t^
Gluteo-sciatic distance	−0.42	0.716 ^t^	1.15	0.322 ^t^	1.46	0.152 ^U^	1.28	0.197 ^t^
Pectineal line length	−1.09	0.337 ^t^	0.80	0.518 ^t^	−1.28	0.286 ^U^	−0.14	0.919 ^t^
Lunate–ramus distance	0.89	0.342 ^t^	1.24	0.207 ^t^	2.05	0.015 ^t^	2.44	0.004 ^t^
Greater sciatic–acetabular distance	2.89	<0.001 ^U^	2.14	0.027 ^U^	2.76	0.003 ^U^	3.03	0.002 ^U^
Lesser sciatic–acetabular distance	1.30	0.214 ^U^	1.07	0.140 ^U^	0.94	0.309 ^U^	0.87	0.341 ^U^
Pubic symphysis height	3.74	<0.001 ^t^	3.42	<0.001 ^t^	3.34	<0.001 ^t^	3.33	<0.001 ^t^
Pubic symphysis width	1.87	<0.001 ^t^	2.00	<0.001 ^t^	1.67	<0.001 ^U^	1.93	<0.001 ^t^
Greater sciatic notch height	1.00	0.142 ^t^	0.25	0.709 ^t^	0.24	0.764 ^t^	0.10	0.874 ^t^
Anterior interspinal height	0.99	0.022 ^U^	0.70	0.040 ^t^	0.75	0.001 ^t^	0.64	0.048 ^U^
Posterior interspinal height	−0.27	0.538 ^U^	0.28	0.942 ^U^	0.42	0.888 ^U^	0.40	0.399 ^U^
Greater sciatic notch angle	−1.91	0.135 ^t^	−0.89	0.496 ^t^	1.61	0.404 ^t^	1.96	0.292 ^t^
Ischiopubic angle	0.98	0.256 ^t^	2.10	0.011 ^t^	1.19	0.161 ^t^	2.06	0.022 ^U^
Ischial angle	−0.61	0.472 ^t^	−0.02	0.864 ^U^	0.70	0.519 ^t^	1.89	0.037 ^t^

*—(age group ≤ 45 years–age group >45 years); t—Welch *t*-test; U—Mann–Whitney U-test.

## Data Availability

The data presented in the study are available on request from the corresponding author.

## Supplementary Materials

The following supporting information can be downloaded at: https://www.mdpi.com/article/10.3390/biology14070866/s1, Table S1: Individual TEM and rTEM values for each measurement; Table S2: Descriptive statistics of the interlandmark distances of the male and female left coxal bones; Table S3: Descriptive statistics of the interlandmark distances of the male and female right coxal bones; Table S4: Significance of sex differences in the interlandmark distances of the left coxal bones; Table S5: Significance of sex differences in the interlandmark distances of the right coxal bones; Table S6: Significance of bilateral differences in the interlandmark distances; Table S7: Descriptive statistic of the first dataset measurements by age; Table S8: Significance of age differences in the interlandmark distances by sex and side; Table S9: Selected features by L1 regularization and RFECV; Table S10: Performance metrics of the models; File S11: Trained models.
