# Supplementary material for: Evaluating the Effectiveness of Coxal Bone Measurements for Sex Estimation via Machine Learning
Source: Biology (Basel). 2025 Jul 17;14(7):866. doi: 10.3390/biology14070866 (PMC12292766; doi:10.3390/biology14070866)
Supplement: Supplementary file 1 [file biology-14-00866-s001.zip › Table S10 Performance metrics od the models.pdf]

Performance metrics of the logistic regression models trained on the first dataset

Model		Train accuracy	Test accuracy	Recall	Precision	F1-score
LR-L2	Right	96.9 ± 2.4 (96.2–97.6)[#]	98.0	F: 100.0<br>M: 96.0	F: 96.0<br>M: 100.0	F: 98.0<br>M: 98.0
	Left	96.7 ± 2.3 (96.1–97.4)	96.0	F: 100.0<br>M: 92.0	F: 93.0<br>M: 100.0	F: 96.0<br>M: 96.0
	Right + Left	97.8 ± 1.4 (97.4–98.2)	96.0	F: 94.0<br>M: 98.0	F: 98.0<br>M: 94.0	F: 96.0<br>M: 96.0
LR-L1	Right	96.7 ± 2.8 (95.9–97.5)	96.0	F: 100.0<br>M: 92.0	F: 93.0<br>M: 100.0	F: 96.0<br>M: 96.0
	Left	96.5 ± 2.4 (95.8–97.1)	96.0	F: 100.0<br>M: 92.0	F: 93.0<br>M: 100.0	F: 96.0<br>M: 96.0
	Right + Left	97.0 ± 1.7 (96.5–97.5)	95.0	F: 92.0<br>M: 98.0	F: 98.0<br>M: 92.0	F: 95.0<br>M: 95.0

[#] - 95% confidence interval; F – females; M - males

Performance metrics of the logistic regression models trained on the second dataset

Model		Train accuracy	Test accuracy	Recall	Precision	F1-score
LR-L2	Right	98.9 ± 1.2 (98.5–99.2)	100.0	F: 100.0<br>M: 100.0	F: 100.0<br>M: 100.0	F: 100.0<br>M: 100.0
	Left	99.0 ± 1.2 (98.6–99.3)	98.0	F: 96.0<br>M: 100.0	F: 100.0<br>M: 96.0	F: 98.0<br>M: 98.0
	Right + Left	99.2 ± 0.9 (98.9–99.4)	99.0	F: 98.0<br>M: 100.0	F: 100.0<br>M: 98.0	F: 99.0<br>M: 99.0
LR-L1	Right	98.1 ± 1.4 (97.8–98.5)	100.0	F: 100.0<br>M: 100.0	F: 100.0<br>M: 100.0	F: 100.0<br>M: 100.0
	Left	99.1 ± 1.1 (98.8–99.4)	98.0	F: 96.0<br>M: 100.0	F: 100.0<br>M: 96.0	F: 98.0<br>M: 98.0
	Right + Left	99.3 ± 0.7 (99.1–99.5)	99.0	F: 98.0<br>M: 100.0	F: 100.0<br>M: 98.0	F: 99.0<br>M: 99.0

[#] - 95% confidence interval; F – females; M - males

Performance metrics of the SVM models trained on the first dataset

Model		Train accuracy	Test accuracy	Recall	Precision	F1-score
SVM	Right	97.3 ± 2.1 (0.967–0.979)[#]	96.0	F: 96.0 M: 96.0	F: 96.0 M: 96.0	F: 96.0 M: 96.0
	Left	97.3 ± 2.0 (96.7–97.9)	96.0	F: 96.0 M: 96.0	F: 96.0 M: 96.0	F: 96.0 M: 96.0
	Right + Left	97.7 ± 1.4 (97.3–98.1)	98.0	F: 96.0 M: 100.0	F: 100.0 M: 96.0	F: 98.0 M: 98.0
SVM RFECV	Right	96.1 ± 2.8 (95.3–96.9)	98.0	F: 100.0 M: 96.0	F: 96.0 M: 100.0	F: 98.0 M: 98.0
	Left	97.3 ± 2.2 (96.7–97.9)	98.0	F: 100.0 M: 96.0	F: 96.0 M: 100.0	F: 98.0 M: 98.0
	Right + Left	97.8 ± 1.3 (97.4–98.2)	98.0	F: 96.0 M: 100.0	F: 100.0 M: 96.0	F: 98.0 M: 98.0

[#] - 95% confidence interval; F – females; M - males

Performance metrics of the SVM models trained on the second dataset

Model		Train accuracy	Test accuracy	Recall	Precision	F1-score
SVM	Right	98.9 ± 1.5 (98.4–99.3)[#]	100.0	F: 100.0 M: 100.0	F: 100.0 M: 100.0	F: 100.0 M: 100.0
	Left	99.1 ± 1.1 (98.8–99.4)	100.0	F: 100.0 M: 100.0	F: 100.0 M: 100.0	F: 100.0 M: 100.0
	Right + Left	99.4 ± 0.7 (99.2–99.6)	99.0	F: 98.0 M: 100.0	F: 100.0 M: 98.0	F: 99.0 M: 99.0
SVM RFECV	Right	99.0 ± 1.2 (98.7–99.4)	100.0	F: 100.0 M: 100.0	F: 100.0 M: 100.0	F: 100.0 M: 100.0
	Left	99.1 ± 1.2 (98.7–99.4)	100.0	F: 100.0 M: 100.0	F: 100.0 M: 100.0	F: 100.0 M: 100.0
	Right + Left	99.3 ± 0.8 (99.1–99.5)	99.0	F: 98.0 M: 100.0	F: 100.0 M: 98.0	F: 99.0 M: 99.0

[#] - 95% confidence interval; F – females; M - males
