# Supplementary material for: Evaluating the Effectiveness of Coxal Bone Measurements for Sex Estimation via Machine Learning
Source: Biology (Basel). 2025 Jul 17;14(7):866. doi: 10.3390/biology14070866 (PMC12292766; doi:10.3390/biology14070866)
Supplement: Supplementary file 1 [file biology-14-00866-s001.zip › Table S9 Selected features by L1 regularization and RFECV.pdf]

LR-L1 Selected Features with Non-Zero Coefficients from the first dataset

Right + Left (27 features)	Left (22 features)	Right (24 features)
mla-iit	mla-iit	mla-iit
sps-pss	ass-pss	ass-pss
ass-pss	asa-ai	ais-pis
ais-pis	ap-lss	asa-ai
mla-mli	sfo-ifo	ap-lss
asa-ai	pirt-is	sfo-ifo
sfo-ifo	gsn-lsn	pirt-is
pirt-is	pss-pis	ips-iipr
ips-iipr	ais-gsn	gsn-lsn
lit-iipr	ais-sij	pss-pis
gsn-lsn	pis-sij	ais-gsn
pss-pis	pgl-gsn	ais-sij
ais-gsn	mipr-lsi	pis-sij
pis-sij	gsn-ap	pgl-gsn
ap-iipr	lsn-ai	pla-plp
pgl-gsn	sps-ips	mipr-lsi
pla-plp	maps-mpps	gsn-ap
gsn-ap	gsn_pirt-is	maps-mpps
lsn-ai	ain_ass-ais	gsn_pirt-is
sps-ips	pin_pss-pis	ain_ass-ais
maps-mpps	pirt-gsn-is	pin_pss-pis
gsn_pirt-is	sps-ips-iipr	pirt-gsn-is
ain_ass-ais		sps-ips-iipr
pin_pss-pis		mipr-iit-lit
pirt-gsn-is		
sps-ips-iipr		
mipr-iit-lit		

LR-L1 Selected Features with Non-Zero Coefficients from the second dataset

Right + Left (28 features)	Left (83 features)	Right (63 features)
ai-mla	ai-ap	ai-as
ap-ifo	ai-iit	ai-asa
as-iipr	ai-mla	ai-ifo
as-is	ai-mpps	ai-mla
as-plp	ain-mla	ain-is
asa-ifo	ais-ap	ain-lit
asa-iipr	ais-ifo	ais-ap
asa-is	ais-iipr	ais-gsn
asa-lit	ais-iit	ais-lit
ass-mla	ais-is	ais-maps
ifo-mla	ais-lsn	ais-mla
iipr-lfo	ais-mla	ap-asa
iipr-lss	ap-as	ap-pirt
iipr-mla	ap-ifo	ap-pis
iit-mla	ap-iit	as-ips
ips-mfo	ap-lsi	as-is
is-pis	ap-mipr	as-lit
lfo-mipr	ap-pgl	as-mla
lfo-mpps	ap-pis	asa-gsn
lit-mla	as-mla	asa-ifo
lsi-mpps	as-plp	asa-is
lsn-mpps	asa-gsn	asa-lit
lss-plp	asa-ifo	asa-mla
maps-sfo	asa-iit	ass-mla
mfo-mipr	asa-is	ifo-lss
mpps-sfo	asa-lit	ifo-mla
pirt-sij	asa-lsi	ifo-sfo
pla-sps	asa-lsn	iipr-lsi
	asa-mla	iit-ips
	asa-plp	iit-is
	ass-mla	ips-lsi
	gsn-ifo	ips-mfo
	gsn-iit	ips-pla
	gsn-lsn	ips-sfo
	gsn-mli	is-lss
	gsn-pgl	is-mipr
	ifo-is	is-pis
	ifo-lfo	is-sfo
	ifo-lss	lfo-mipr
	ifo-mla	lfo-sfo
	iipr-lfo	lit-mla
	iipr-mfo	lit-mpps
	iipr-sfo	lsi-mli
	iit-is	lsi-mpps
	iit-mla	lsi-pis
	ips-lfo	lsn-mpps
	ips-lsi	maps-pla
	ips-lsn	maps-sfo
	ips-mfo	mfo-mipr
	ips-pla	mla-mli
	ips-sfo	mla-sij
	is-lsn	mli-sij
	is-mfo	mpps-pla

	is-mipr<br>is-pgl<br>is-pirt<br>is-pis<br>is-sfo<br>lfo-mipr<br>lfo-mla<br>lfo-mpps<br>lfo-sps<br>lit-mla<br>lit-mpps<br>lsi-mla<br>lsi-mpps<br>lsi-sps<br>lsn-mla<br>lsn-mpps<br>lss-mipr<br>lss-plp<br>lss-sfo<br>mfo-mipr<br>mfo-mpps<br>mla-plp<br>mpps-pla<br>mpps-plp<br>mpps-sfo<br>pin-pis<br>pirt-sij<br>pis-pss<br>plp-sps<br>sfo-sps	mpps-sfo<br>mpps-sij<br>pin-pis<br>pin-sij<br>pirt-sij<br>pis-sij<br>pla-sps<br>plp-sij<br>sfo-sps<br>sij-sps

RFECV Selected Features from the first dataset

Right + Left	Left	Right
Optimal number of features: 22<br>Selected feature:<br>'mla-iit', 'sps-pss', 'ass-pss', 'mla-mli', 'asa-ai', 'ap-lss', 'sfo-ifo', 'pirt-is', 'ips-iipr', 'lit-iipr', 'gsn-lsn', 'pss-pis', 'ais-gsn', 'ais-sij', 'pis-sij', 'ap-iipr', 'pgl-gsn', 'mipr-lsi', 'lsn-ai', 'maps-mpps', 'pirt-gsn-is', 'sps-ips-iipr'	Optimal number of features: 27<br>Selected feature:<br>'mla-iit', 'sps-pss', 'ass-pss', 'mla-mli', 'asa-ai', 'ap-lss', 'sfo-ifo', 'pirt-is', 'ips-iipr', 'lit-iipr', 'gsn-lsn', 'pss-pis', 'ais-gsn', 'ais-sij', 'pis-sij', 'ap-iipr', 'pgl-gsn', 'mipr-lsi', 'gsn-ap', 'lsn-ai', 'maps-mpps', 'gsn_pirt-is', 'ain_ass-ais', 'pin_pss-pis', 'pirt-gsn-is', 'sps-ips-iipr', 'mipr-iit-lit'	Optimal number of features: 18<br>Selected feature:<br>'mla-iit', 'mla-mli', 'asa-ai', 'ap-lss', 'sfo-ifo', 'pirt-is', 'ips-iipr', 'lit-iipr', 'gsn-lsn', 'ais-gsn', 'pis-sij', 'ap-iipr', 'pgl-gsn', 'mipr-lsi', 'lsn-ai', 'maps-mpps', 'pirt-gsn-is', 'sps-ips-iipr'

RFECV Selected Features from the second dataset

Right + Left	Left	Right
Optimal number of features: 137	Optimal number of features: 60	Optimal number of features: 25
Selected feature:	Selected feature:	Selected feature:
ai-asa	ai-is	ais-is
ai-ips	ai-mla	as-ifo
ai-is	ai-plp	as-iipr
ai-lit	ais-iipr	as-is
ai-mla	ais-iit	asa-ifo
ai-mpps	ais-is	asa-iipr
ai-pla	ap-ifo	asa-is
ain-gsn	ap-lss	iipr-mla
ain-iipr	as-ifo	iipr-plp
ain-iit	as-iipr	iit-mla
ain-lit	as-iit	iit-plp
ain-maps	as-is	ips-lfo
ain-mipr	as-lsn	ips-mfo
ain-mla	asa-ifo	ips-sfo
ais-gsn	asa-iipr	lfo-mpps
ais-iipr	asa-iit	lit-mla
ais-iit	asa-is	lss-plp
ais-is	asa-lsn	maps-sfo
ais-lit	asa-mipr	mipr-pla
ais-mla	gsn-pin	mipr-plp
ais-mli	gsn-pis	mpps-pla
ais-sps	ifo-ips	mpps-sfo
ap-as	ifo-mla	pirt-sij
ap-asa	ifo-plp	pla-sps
ap-ifo	iipr-mla	sfo-sps
ap-iipr	iipr-mli	
ap-iit	iipr-plp	
ap-is	iipr-sfo	
ap-mipr	iit-mla	
ap-sfo	iit-mli	
as-ifo	iit-plp	
as-iipr	ips-lfo	
as-iit	ips-lsi	
as-is	ips-mfo	
as-lit	ips-sfo	
as-lsn	is-lss	
as-plp	is-plp	
asa-ifo	is-sfo	
asa-iipr	lfo-maps	
asa-iit	lfo-mpps	
asa-is	lit-mla	
asa-lit	lit-mpps	
asa-lsi	lsi-maps	
asa-lsn	lsi-mla	
asa-mipr	lsi-mpps	
asa-mla	lsn-mla	
asa-mli	lsn-mpps	
asa-mpps	lss-mpps	
gsn-ifo	lss-plp	
gsn-iit	maps-mfo	
gsn-pin	maps-sfo	

gsn-pis<br>ifo-ips<br>ifo-lsi<br>ifo-lss<br>ifo-mla<br>ifo-mpps<br>ifo-plp<br>iipr-lfo<br>iipr-lsi<br>iipr-lss<br>iipr-mla<br>iipr-mli<br>iipr-pla<br>iipr-plp<br>iit-is<br>iit-lsi<br>iit-lsn<br>iit-mla<br>iit-mli<br>iit-plp<br>ips-lfo<br>ips-lit<br>ips-lsi<br>ips-lsn<br>ips-lss<br>ips-mfo<br>ips-mipr<br>ips-sfo<br>is-lss<br>is-mipr<br>is-mla<br>is-mpps<br>is-pin<br>is-pirt<br>is-pis<br>is-plp<br>is-sfo<br>lfo-maps<br>lfo-mipr<br>lfo-mla<br>lfo-mpps<br>lfo-plp<br>lfo-sps<br>lit-mla<br>lit-mli<br>lit-mpps<br>lit-pgl<br>lit-pis<br>lit-sfo<br>lsi-maps<br>lsi-mla<br>lsi-mpps<br>lsi-pis<br>lsi-sps<br>lsn-mla<br>lsn-mpps	mfo-mpps<br>mipr-mla<br>mipr-pla<br>mli-sij<br>mpps-pla<br>mpps-sfo<br>pirt-sij<br>pla-sps<br>sfo-sps	

lss-maps<br>lss-mla<br>lss-mpps<br>lss-plp<br>maps-mfo<br>maps-mpps<br>maps-pla<br>maps-sfo<br>mfo-mipr<br>mfo-mpps<br>mipr-mla<br>mipr-pla<br>mipr-plp<br>mla-sfo<br>mli-mpps<br>mli-sij<br>mpps-pin<br>mpps-pirt<br>mpps-pis<br>mpps-pla<br>mpps-plp<br>mpps-sfo<br>mpps-sij<br>pgl-sij<br>pin-sij<br>pirt-sij<br>pis-pss<br>pla-sps<br>pss-sps<br>sfo-sps		
